# Promoting Healthy and Supportive Acoustic Environments: Going beyond the Quietness

**DOI:** 10.3390/ijerph16244988

**Published:** 2019-12-08

**Authors:** Francesco Aletta, Jian Kang

**Affiliations:** UCL Institute for Environmental Design and Engineering, The Bartlett, University College London (UCL), Central House, 14 Upper Woburn Place, WC1H 0NN London, UK

When confronted with the topic of the quality of the acoustic environments, society and communities around the world tend to consider “sound” mainly in its negative facet of “noise”. This approach is reflected in a number of recommendations and prescriptions to reduce people’s exposure to excessive sound levels from transportation and industry, promoted by international institutions and authorities, such as the World Health Organization or the European Union [1,2]. Notwithstanding, such a strategy is not always effective in delivering the desired enhancements in terms of health and quality of life, and this is because “quietness” and the pursuit of “silence” are not necessarily enough to define an acoustic environment of high quality [3]. Indeed, environmental sounds often have positive effects on people, as they provide information, communicate safety, enable certain desirable activities, and, more generally, contribute to people’s appeasement and psychophysical well-being [4,5]. With the rapid increase of urbanization, more research is needed towards alternative approaches for the characterization, management, and design of urban acoustic environments that support (and not only allow) restoration, health, and better quality of life, as well as basic research on the mechanisms underpinning the perception of environmental sounds in context and how their experience might affect health-related outcomes.

Researchers in the environmental acoustics and soundscape domains are addressing these challenges by exploring new inter- and trans-disciplinary approaches to the characterization of the quality of the acoustic environments, new prediction and modeling methodologies for the acoustic environments and their qualities, and the relationships between sound, space, and behaviors in the built environment. From the 16 contributions published in this Special Issue, three main research themes were identified, which are briefly discussed below.

## 1. Rethinking Quiet Areas and Their Restorative Potential for Health and Well-Being 

The concept of perceived quietness has been thoroughly investigated over the years, partly also due to the emphasis put on this topic by major environmental agencies and policymakers. However, clear criteria that go beyond setting a mere sound level threshold for identifying areas where quietness can be experienced have not been internationally agreed on so far. The connection with natural elements and the experience of greenery seem to be prerequisites for quietness and similarly related perceptual constructs (e.g., tranquillity, calmness), and this applies at different urban scales; however, many aspects still need to be clarified, as some of the contributions of this Special Issue highlighted.

Dzhambov et al. [6] show that more green areas in residential environments are associated with lower noise annoyance, whereas higher tree cover is similarly effective only in small-radius buffer zones. Hedblom and colleagues [7] question a relatively well-established concept in soundscape studies, that birdsong typically facilitates stress recovery, by reporting that in their listening experiment, such effect was, in fact, not observed. This more generally raises the point about what sound sources one would expect to contribute more to quietness perception, as well as supporting health and well-being. To some extent, the work by Payne and Bruce [8] also claims that the type of sounds heard and other aspects of a site experience are likely to be related to a non-linear relationship between sound levels and perceived restoration of an acoustic environment. Other contextual aspects might indeed play a role, such as audio–visual interactions in environmental perception, as suggested in the work by Zhang et al. [9]. Cerwén [10] explores new autoethnographic approaches to propose possible actions, which are organized into three main categories (i.e., localization of functions, reduction of unwanted sounds, and introduction of wanted sounds) that designers can take into account when managing quiet areas in the urban realm. Finally, Cerwén and Mossberg [11] report on a study about the implementation of quiet areas in accordance with the EU Environmental Noise Directive (END) of 2002, conducted with several municipalities in Sweden. They find out that at the local authority level, many initiatives have dealt with mapping and identification of quiet areas, but less has been done regarding their maintenance and enforcement, and this is likely to be a common issue for other (European) countries too.

## 2. Extending the Research Scope to More Soundscape Quality Dimensions, Contextual, and Physiological Factors 

While quietness is certainly a key theme in the current discourse about community noise, other dimensions might be relevant in different contexts to characterize the acoustic quality of public spaces. It is fair to assume that non-quiet spaces might still have the potential to promote positive user experiences of an urban environment, or that quietness cab not necessarily always match less “loud” acoustic environments. Herranz-Pascual and colleagues [12] indeed suggest that lively and vibrant urban soundscapes may also enhance people’s restoration. Aletta and Kang [13] propose a model to predict urban “vibrancy” using a soundscape approach. Perceived vibrancy can be predicted by a set of (psycho)acoustic parameters, the number of visible people on site, and the presence of music in the auditory scene. Music is indeed a key component of modern urban soundscapes: Whether designed or not, it has the potential to enhance the social experience of a place. Steele and colleagues [14] carried out an interventional study with an unsupervised installation in a public pocket park allowing users to play audio content from personal devices over publicly provided speakers and they found out that the soundscape was experienced as more pleasant for both users and non-users, and the calmness and appropriateness of the place were not affected. Xiao and Hilton [15] investigate the factors influencing sound environments perception in relation to “square dancing”, a growing social phenomenon in many Chinese cities. A better understanding of contextual factors and aspects related to the interactions between people and places is required to characterize soundscapes holistically, as pointed out by Hermida and colleagues [16] in their case studies in Lisbon and Bogotá. For this purpose, it is certainly useful to review the corpus of literature looking at the positive effects that soundscapes and environmental sounds more generally can have on people’s quality of life and well-being [17,18].

## 3. Supportive Indoor Soundscapes—A Perceptual Perspective on Building and Room Acoustics 

The quality of outdoor acoustic environments is, of course, a mainstream dimension in the narrative of soundscape studies. However, considering that people spend the vast majority of their time in indoor environments, addressing the acoustic quality of such spaces is of paramount importance. It is, therefore, right to question how we expect buildings to “sound like” in order to promote supportive indoor soundscapes. While the acoustics in buildings has typically been dealing with sound insulation and room acoustics performances, a new perceptual perspective on the topic is gradually finding its way in. Taghipour et al. [19] look at how different building facades are likely to affect the perceived acoustic quality of inner yards of residential complexes. Di Blasio and colleagues [20] focus on the overall comfort and performance of open-plan office workers when affected by undue noise coming from irrelevant speech. Similarly, looking at cognitive performance, Shu and Ma [21] carry out a study to explore the restorative effects of different soundscapes on children’s sustained attention and short-term memory.

Overall, the works published in this special issue reflect an active and engaged research community, which is exploring the connections between human well-being and the acoustic quality of the built environment from a broad range of methodologies and perspectives in terms of quietness and other dimensions. This is promising, as more efforts are required to address the challenges we face in promoting positive acoustic environments beyond noise control and acoustically “sanitized” spaces.
